# Comparison of PET template-based and MRI-based image processing in the quantitative analysis of C^11^-raclopride PET

**DOI:** 10.1186/2191-219X-4-7

**Published:** 2014-01-22

**Authors:** Felix P Kuhn, Geoffrey I Warnock, Cyrill Burger, Katharina Ledermann, Chantal Martin-Soelch, Alfred Buck

**Affiliations:** 1 Department of Nuclear Medicine, University Hospital Zurich, Rämistrasse 100, Zurich 8091, Switzerland; 2 Department of Psychiatry and Psychotherapy, University Hospital Zurich, Culmannstrasse 8, Zurich 8091, Switzerland; 3 Department of Clinical and Health Psychology, University of Fribourg, Rue P-A Faucigny 2, Fribourg 1700, Switzerland; 4 PMOD Technologies Ltd., Sumatrastrasse 25, Zurich 8006, Switzerland

**Keywords:** Neuroimaging, PET/MRI, Raclopride, Movement disorders

## Abstract

**Background:**

Quantitative measures of ^11^C-raclopride receptor binding can be used as a correlate of postsynaptic D_2_ receptor density in the striatum, allowing ^11^C-raclopride positron emission tomography (PET) to be used for the differentiation of Parkinson’s disease from atypical parkinsonian syndromes. Comparison with reference values is recommended to establish a reliable diagnosis. A PET template specific to raclopride may facilitate direct computation of parametric maps without the need for an additional MR scan, aiding automated image analysis.

**Methods:**

Sixteen healthy volunteers underwent a dynamic ^11^C-raclopride PET and a high-resolution T1-weighted MR scan of the brain. PET data from eight healthy subjects was processed to generate a raclopride-specific PET template normalized to standard space. Subsequently, the data processing based on the PET template was validated against the standard magnetic resonance imaging (MRI)-based method in 8 healthy subjects and 20 patients with suspected parkinsonian syndrome. Semi-quantitative image analysis was performed in Montreal Neurological Institute (MNI) and in original image space (OIS) using VOIs derived from a probabilistic brain atlas previously validated by Hammers et al. (Hum Brain Mapp, 15:165–174, 2002).

**Results:**

The striatal-to-cerebellar ratio (SCR) of ^11^C-raclopride uptake obtained using the PET template was in good agreement with the MRI-based image processing method, yielding a Lin’s concordance coefficient of 0.87. Bland-Altman analysis showed that all measurements were within the ±1.96 standard deviation range. In all 20 patients, the PET template-based processing was successful and manual volume of interest optimization had no further impact on the diagnosis of PD in this patient group. A maximal difference of <5% was found between the measured SCR in MNI space and OIS.

**Conclusions:**

The PET template-based method for automated quantification of postsynaptic D_2_ receptor density is simple to implement and facilitates rapid, robust and reliable image analysis. There was no significant difference between the SCR values obtained with either PET- or MRI-based image processing. The method presented alleviates the clinical workflow and facilitates automated image analysis.

## Background

Positron emission tomography (PET) with ^11^C-raclopride is used for the differentiation of Parkinson’s disease (PD) from atypical parkinsonian syndromes [1]. In early stages of PD, the postsynaptic D_2_ receptor density is upregulated whereas in atypical parkinsonian syndromes, i.e. multiple system atrophy (MSA), progressive supranuclear palsy (PSP) and corticobasal degeneration (CBD), a normal or decreased receptor density is found [2]. As a correlate of postsynaptic D_2_ receptor density in the putamen and caudate nucleus (striatum), quantitative measures of ^11^C-raclopride binding, combined with comparison to reference values, are recommended for reliable diagnosis [3].

Region-based methods for PET quantification are widely used and have been shown to yield reliable results [4]. However, manual delineation of regions of interest (ROIs) or volumes of interest (VOIs) are user-dependent and time-consuming. Furthermore, these techniques introduce intra- and interoperator variation, which limits the reproducibility of the results. Several atlas-based methods function through normalization of corresponding MR images into a stereotaxic spatial array and application of the same transformation to the co-registered PET images [5]. A challenge often encountered is that the normalization of subcortical regions and particularly the striatum is prone to errors, mainly due to differences in the morphology of the ventricular system. Thus, subcortical VOIs often had to be user-adjusted. Furthermore, where PET tracer uptake is limited to discrete regions, as for ^11^C-raclopride, co-registration of the PET image to the corresponding MR image may be inaccurate. Dynamic scanning can provide perfusion-like data immediately after tracer injection which can be used for more accurate PET to MR co-registration, at the cost of increased scan time [6,7]. Advanced magnetic resonance imaging (MRI)-based VOI definition methods use computer vision techniques for the segmentation of the desired structures. Yasuno et al. [8] utilized MR images to determine the probabilities of grey matter to refine the ROIs. Svarer et al. [9] reduced the individual variability by applying a warping algorithm to several segmented brains to estimate probabilistic ROIs for an individual brain. An even more sophisticated technique was applied by Rusjan et al. [10], using the Montreal Neurological Institute/International Consortium of Brain Mapping standard brain template (MNI/ICBM), segmentation using a fitting function of grey matter probability and multiple iterations of morphological dilatation to prevent overlap between neighbouring ROIs [10].

A number of approaches based on ligand-specific templates have already been assessed with promising results for further development of automated data processing and image analysis [11-13]. However, there is an ongoing discussion if measurements made in the MNI space are as reliable as the measurement made in the subject’s original image space (OIS), as different transformations and interpolations have been applied to the image data. A raclopride template could make direct computation of parametric maps in standard or original imaging space possible without the need for early time frames in PET and an additional MR scan. This would facilitate efficient clinical workflow and automated image analysis.

## Methods

A total of 16 healthy volunteers (mean age 45, range 25 to 70; all females) and 20 consecutive patients with suspected parkinsonian syndrome (mean age 60, range 45 to 81; 9 females, 11 males) were included. All volunteers underwent a dynamic ^11^C-raclopride PET (0 to 60 min postinjection (p.i.)) and a high-resolution MR scan of the brain (details below). All patients underwent a static ^11^C-raclopride PET scan (40 to 60 min p.i.). The study was approved by the institutional ethics committee of the canton of Zurich, Switzerland, and written informed consent was obtained from all participants.

### PET and MR data acquisition

^11^C-raclopride was produced on site according to GMP guidelines. The PET examinations were performed on a full ring PET/CT scanner with an axial field of view of 15.3 cm in three-dimensional (3D) mode (Discovery STE, GE Healthcare, Waukesha, WI, USA). The emission data was corrected for attenuation using the corresponding CT (120 kV/80 mA). The PET images were reconstructed using a standard iterative algorithm (ordered set expectation maximization (OSEM)) by integrating further correction algorithms to reduce image noise caused by scatters and randoms. The dynamic PET scan was started immediately after bolus injection of 250 MBq ^11^C-raclopride, and emission data was acquired over 60 min with frame durations of 1 min (0 to 15 min p.i.) and 5 min (15 to 60 min p.i.). Static PET acquisition was performed 40 to 60 min after tracer injection. Volumetric MR data was acquired in the transverse plane using a T1-weighted 3D gradient echo MR pulse sequence with isotropic voxels (1 mm). MR scan parameters were as follows: field strength, 3 T; time of repetition (TR), 8.8 ms; time of echo (TE), 2.3 ms; flip angle, 8° and number of excitations (NEX), 1.

### Image processing and analysis

**Figure 1:**
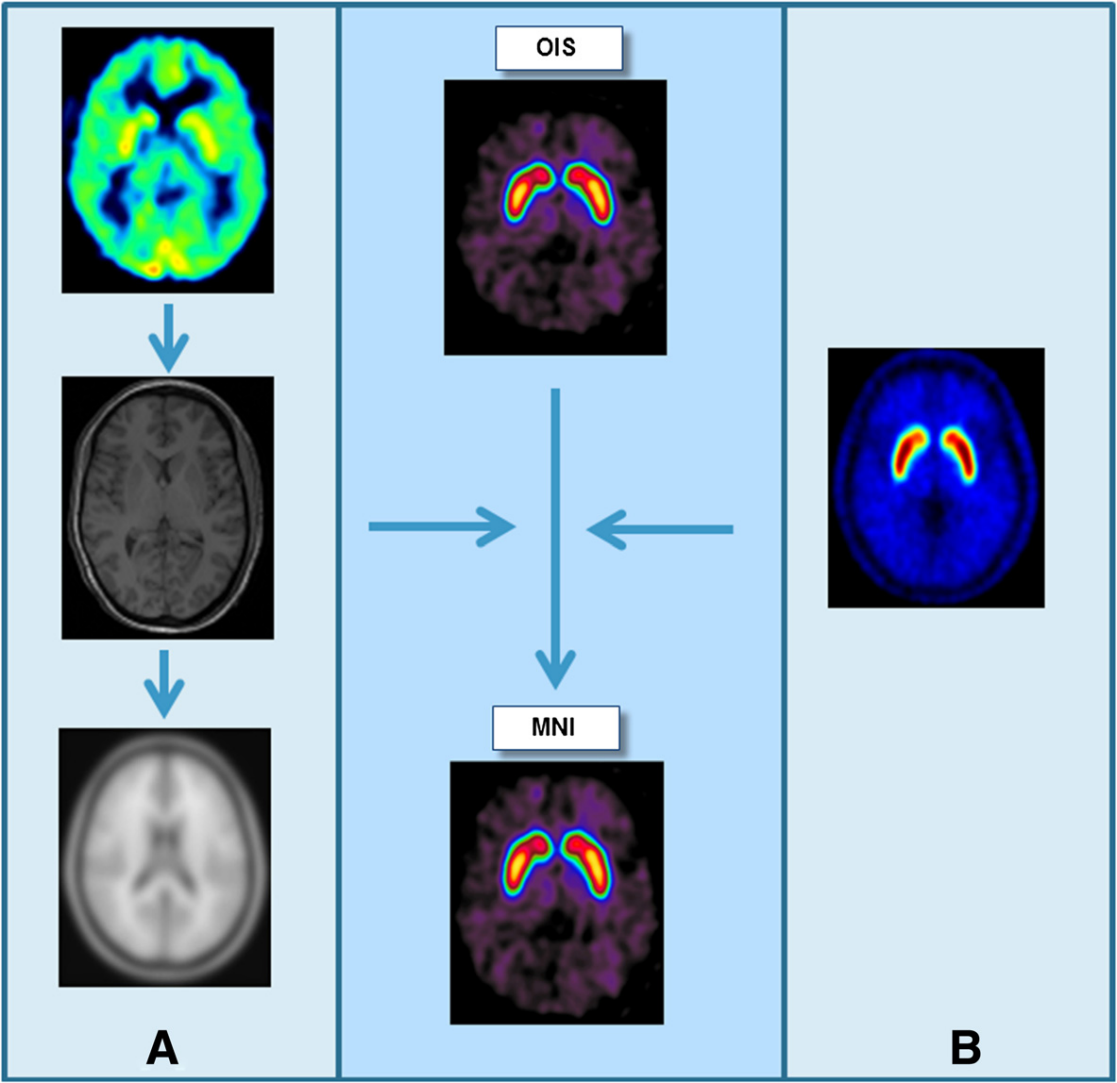
**Normalization of the binding PET images. (A)** Rigid co-registration of perfusion-like PET images to MRI (transformation matrix t1). Spatial normalization of MR images in original image space (OIS) into MNI space (t2). Combination of t1 and t2 to normalize the binding PET images into the MNI space. **(B)** Direct transformation of binding PET images to MNI space by normalization to the raclopride PET template.

All data processing was performed using PMOD Version 3.4 (PMOD Technologies Ltd., Zurich, Switzerland). Two approaches to spatial normalization were investigated: MRI-based and PET template-based (Figure 1). The early PET frames (1 to 8 min p.i.) reflecting cerebral blood flow were averaged to obtain a *perfusion-like image*. Individual *raclopride binding images* from healthy volunteer dynamic data were generated by averaging the PET frames acquired between 40 and 60 min after tracer injection. Averaging of multiple frames was performed to obtain images with a high signal-to-noise ratio (SNR). Motion correction based on a reference frame (10 to 15 min p.i.) was performed on all dynamic PET frames to correct for minor subject movements during the emission scan.

#### MRI-based spatial normalization

*Rigid image co-registration of PET to MRI*. The perfusion-like images generated from dynamic data in healthy volunteers outlining the cortical and subcortical grey matter were co-registered to the corresponding 3D T1w MR images by applying a normalized mutual-information-based rigid registration. The resulting transformation matrix was applied to the corresponding binding images.

*Spatial normalization of MRI*. Normalization of the MRI images to a T1 MR template image matching the MNI stereotaxic space (identical to the T1 template provided with SPM5, derived from the ICBM152 image) was performed using an SPM5 compliant procedure with 16 iterations and a cutoff of 25.

*Spatial normalization of PET*. The transformation matrices of the rigid registration of PET to MRI and the elastic registration of MRI to stereotaxic MNI space were combined to normalize the PET binding images into the MNI space.

#### PET template-based spatial normalization

*Generation of the PET template*. Spatially normalized binding images from eight independent healthy subjects were averaged to obtain a high SNR raclopride PET template. To ensure that the eight subjects contributed equally to the final template, the mean activity in the basal ganglia was normalized to a value of 1.

*Spatial normalization of PET images to raclopride PET template*. Raclopride binding images were directly transformed into MNI space by normalization to the raclopride PET template with the same parameters as described for the MRI normalization.

#### Quantitative VOI analysis

Following normalization, the maximum probability atlas (Hammers N30R83) was intersected with the 50% grey matter probability map and used for VOI definition. The construction of the N30R83 atlas and the segmentation algorithm has been described elsewhere [5,14].

**Figure 2:**
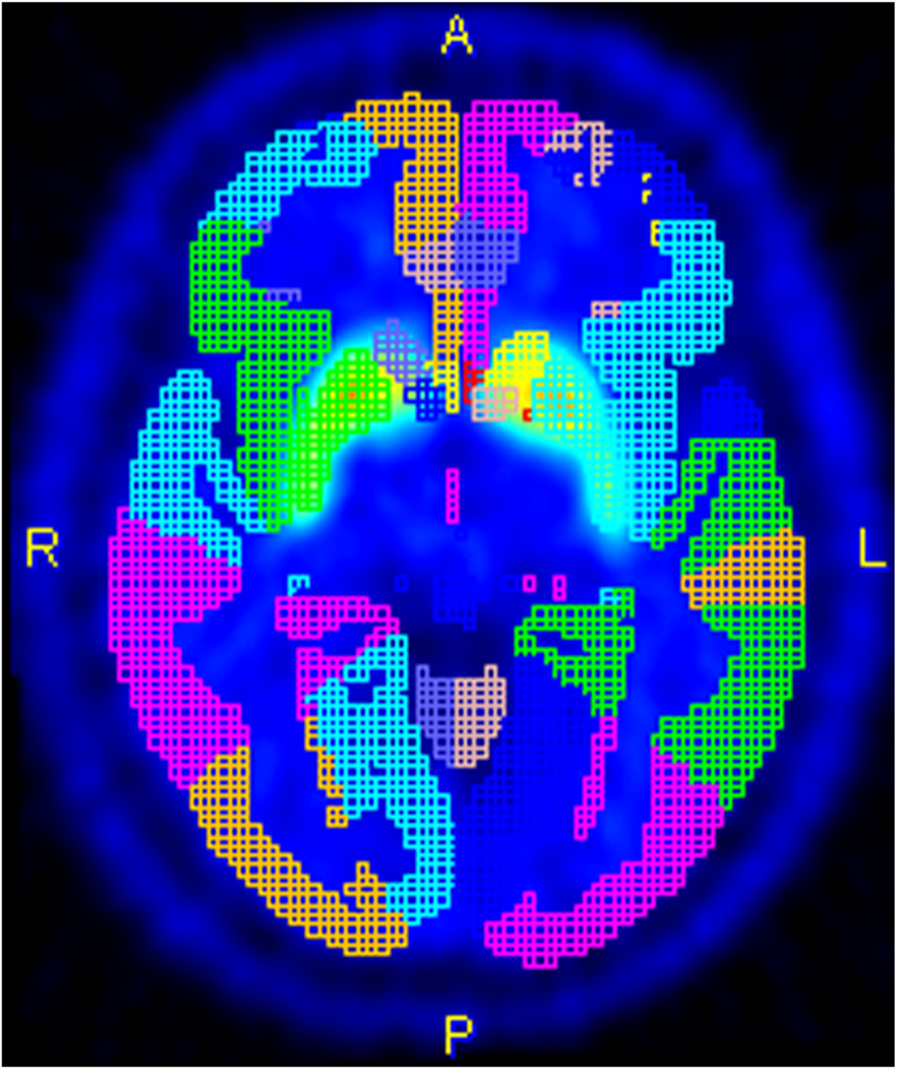
Maximum probability atlas (Hammers N30R83) for VOI definition.

Subsequently, the measured radioactivity in all voxels was divided by the mean activity in the cerebellum (reference region) to standardize for levels of free tracer. ^11^C-raclopride uptake in striatal VOIs was quantified, including the caudate nucleus and the putamen (Figure 2). The analysis was performed in both MNI space and the subject’s OIS, due to potential concerns regarding the correctness of the measurements after data transformation and interpolation during the spatial normalization procedure. The atlas-based VOIs were transformed into OIS using the inverse of the combined transformation matrices. The percentage difference between the activity concentration measured in the MNI space and in OIS was computed according to the following formula:

$$\mathrm{Difference}\%=\left(\frac{\mathrm{SCR}\;\left(\mathrm{MNI}\right)}{\mathrm{SCR}\;\left(\mathrm{OIS}\right)}–1\right)\;\times \;100\%\;$$

where the striatal-cerebellar ratio (SCR) indicates the normalized mean tissue radioactivity concentration in the striatum.

#### Validation with patient data

Static raclopride PET binding images from 20 patients with suspected parkinsonian syndrome were normalized to MNI space using the PET template. Following normalization, the maximum probability atlas (Hammers N30R83) was applied for VOI definition. As a standard of reference, the definition of the atlas VOIs was verified in three orthogonal image planes by an experienced nuclear medicine physicist and manually optimized if necessary. The data was analyzed in both MNI space and OIS.

**Figure 3:**
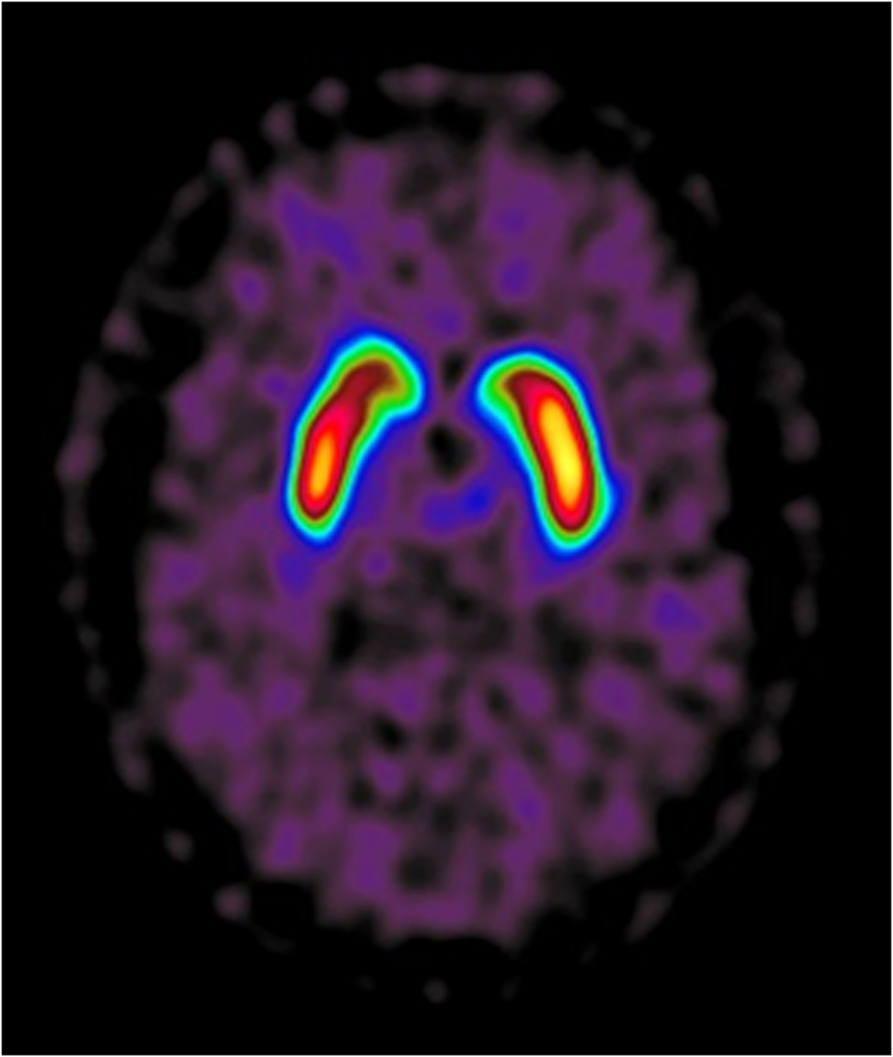
**Patient with parkinsonian syndrome.** With clinical symptoms predominantly on the right side and pronounced increase of raclopride uptake on the contralateral left side. Z score, Caudate nucleus right, 2.5 and left, 3.0. Putamen right, 4.9 and left, 6.0.

Image findings supporting the diagnosis of PD are increased raclopride-uptake contralateral to the dominant clinical symptoms and a high Z-score compared to the reference population (Figure 3).

### Statistical evaluation

Statistical analysis was performed using SPSS (SPSS Version 20, IBM Corporation, Armonk, NY, USA). The degree of agreement between the MR and PET template method was analyzed by using Bland-Altman plots and by calculating the Lin’s concordance coefficient.

For the global statistical assessment, the right and left striatum were analyzed separately. For the comparison of the measurements in MNI and OIS, the caudate nucleus and the putamen from left and right were assessed together.

## Results

All PET and MR images of the 16 healthy subjects were of high quality and were included for further evaluation. In 12 out of the 20 patients, PD was confirmed by raclopride imaging and clinical 1 year follow-up. The other eight patients were diagnosed with essential tremor. No patients with MSA, PSP or CBD were in the study population.

**Figure 4:**
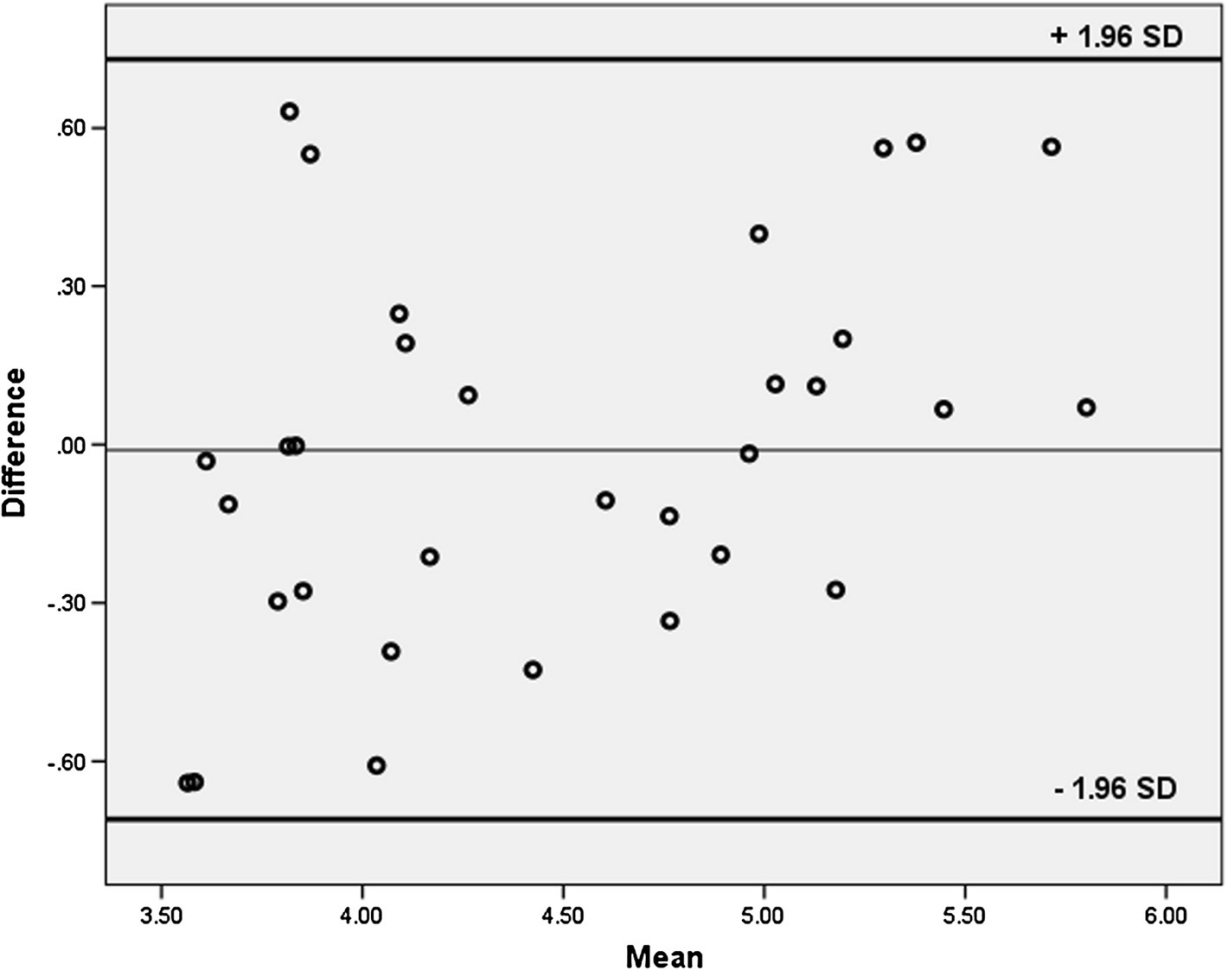
Bland-Altman plot comparing the measurements of the two approaches (SD = 0.36).

Comparing the MR and PET template-based spatial normalization methods, there was a high degree of concordance between the measured tissue concentrations in the caudate nucleus and the putamen in MNI space with a Lin’s concordance coefficient (*ρ*_c_) of 0.87 including an upper one-sided 95% confidence interval of *ρ*_c_ = 0.93 and a lower one-sided 95% confidence interval of *ρ*_c_ = 0.79 [15]. Bland-Altman analysis indicated that all measurements were within the ±1.96 standard deviation range (Figure 4) without evidence of systematic bias (standard deviation 0.36, absolute values for the limits of agreement +0.63 and -0.64).

**Figure 5:**
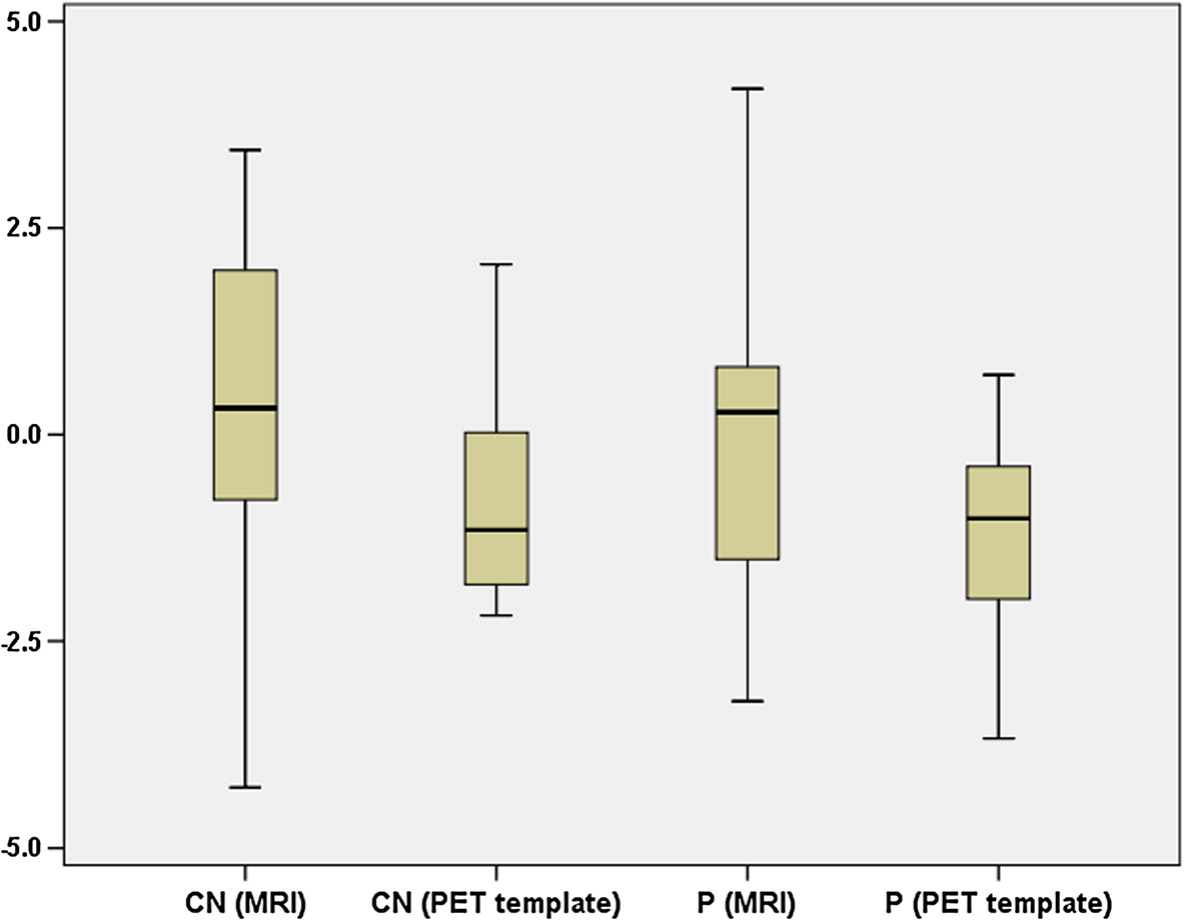
**Percentage difference of quantitative measurement in MNI space and in original image space.** Based on the MRI method or the generated PET template (CN, caudate nucleus; P, putamen).

Based on the MRI approach, the maximal difference between the measurements in MNI space and after back-transformation to the OIS was 4.3% in the caudate nucleus (mean 0.3, standard deviation 2.4) and 4.2% in the putamen (mean 0.2, SD 2.2). The direct approach based on the PET template yielded a maximal difference between the measurements in MNI or OIS of 2.2% in the caudate nucleus (mean -0.8, SD 1.4) and 3.7% in the putamen (mean -1.2, SD 1.4) (Figure 5). No significant difference (paired *t* test) between the measurements in MNI space or OIS was found, with *p* = 0.72.

PET template-based processing was successful in all 20 patients. Image analysis resulted in diagnosis of PD in 12 patients, and further analysis of SCR was only performed in those 12 patients. The maximal difference between measurements with automated VOI placement and after manual VOI optimization (standard of reference) was 4.5% (mean 0.6%, SD 1.9), which had no impact on the diagnosis of PD in this patient subgroup.

## Discussion

A raclopride binding PET template generated using dynamic PET data from healthy volunteers facilitated direct quantification of ^11^C-raclopride binding in the putamen and caudate nucleus (striatum). Quantitative measures of D2 receptor density with comparison to reference values are recommended for the detection of the early stages of movement disorders and establishment of a reliable diagnosis.

Raclopride binding is typically assessed between 40 to 60 min after tracer injection, once free tracer has cleared from the blood pool. However, at this time point, tracer binding is limited to D2 receptor dense regions, namely the caudate nucleus and putamen, making automatic co-registration of the PET data to the corresponding MR highly inaccurate. Perfusion-like images from early time frames of PET imaging are more suitable for image co-registration with morphological MRI [6,7]. However, the need for MR images and full dynamic PET series in addition to receptor binding images from late time frames in PET is not cost-effective in a clinical setting. Extra image processing are necessitated which may be less readily automated.

The sophisticated MR postprocessing technique applied in the present study used a combination of grey matter segmentation, a probabilistic atlas and a mutual information-based normalization process to achieve accurate image co-registration to the MNI/ICBM standard brain template. Automated analysis of quantitative PET data requires robust image processing with a minimum number of opportunities to introduce errors [16]. Techniques based on MRI require high-quality images to ensure correct segmentation and normalization, which is not always straightforward in patients with movement disorders. As manually optimized VOIs are user-dependent and time-consuming, they do not represent an ideal alternative to atlas-based measurements.

The PET raclopride binding template allows for direct computation of SCR parametric maps after a single normalization step. This procedure facilitates automated data processing and image analysis. One-step co-registration of raclopride binding images to the binding template may also reduce the risks of errors introduced in the multi-step MR-based process. Furthermore, user-independent data quantification reduces intra- and interoperator variations which can otherwise limit the reproducibility of the measurements. Direct normalization of the PET data obviates the need for the acquisition of early PET frames in a dynamic protocol and for an additional MR scan. Patients must thus spend less time in the scanner, improving patient comfort and cost-effectiveness in clinical routine. Taken together, the development of a PET binding template and the use of one-step brain normalization suggest that a total image acquisition time of about 10 to 20 min could be sufficient to establish a reliable diagnosis in patients with parkinsonian syndrome.

As an initial validation of the PET-template based image processing protocol, we applied it in a small cohort of patients. Direct image normalization using the PET template was successful in all cases, and further analysis of the PD diagnosed subgroup indicated that quantitative measurements of ^11^C-raclopride binding were within 5% of the values from manually defined VOIs (standard of reference). This level of variation was not sufficient to influence diagnosis of PD and supports the use of static PET ^11^C-raclopride imaging without MR in the clinic.

The calculation of PET VOI statistics can be performed in both the original image space and after normalization to the MNI space. In order to examine the effect of interpolation of the original PET values during transformation, the atlas-based VOIs were back-transformed to the original PET image space (OIS). The maximal difference in SCR between the measurements in MNI template space and in OIS was found to be <5%, suggesting that either measurement should be suitable for the quantification of clinical data. No systemic measurement errors with significantly higher or lower quantitative values in OIS or MNI were found. The slight tendency to higher quantitative measurements in OIS when using the PET template method might be explained by the smoothing and interpolation process during image normalization into MNI space. These results may legitimate the direct measurements in the MNI space which is more easily implemented in an automated image analysis procedure.

In line with a previous study by Meyer et al. [11], we found that the ligand template method provides a reliable approach for spatial normalization of PET ligand images. We confirmed these results by using perfusion-like early raclopride frames to assure the most accurate co-registration of the PET and MRI data and by applying advanced spatial normalization techniques especially suited for the normalization of the basal ganglia. Furthermore, we used an atlas-based approach eliminating the user-dependent and time-consuming VOI delineation.

## Conclusions

A PET template-based method for automated quantification of postsynaptic D_2_ receptor density is simple to implement and allows for rapid, robust and reliable image analysis. There was no significant difference between the SCR values obtained using this method and the more demanding MR-based image processing. The method presented simplifies the clinical workflow and facilitates automated image analysis.

## Competing interests

Cyrill Burger is CEO of PMOD Technologies Ltd. The other authors declare that they have no competing of interests.

## Authors’ contributions

FPK and GIW performed the image processing and analysis and drafted the manuscript. KL and CMS carried out the data acquisition. AB and CB conceived of the study and participated in its design and coordination. All authors read and approved the final manuscript.
